# Effect of Interlayer Construction on TFC Nanofiltration Membrane Performance: A Review from Materials Perspective

**DOI:** 10.3390/membranes13050497

**Published:** 2023-05-08

**Authors:** Mingxiang Liu, Lei Zhang, Nannan Geng

**Affiliations:** School of Civil Engineering and Architecture, Chuzhou University, Chuzhou 239000, China

**Keywords:** thin-film composite, nanofiltration membrane, water permeability, selectivity, water purification

## Abstract

Polyamide (PA) thin-film composite (TFC) nanofiltration (NF) membranes, which are extensively utilized in seawater desalination and water purification, are limited by the upper bounds of permeability-selectivity. Recently, constructing an interlayer between the porous substrate and the PA layer has been considered a promising approach, as it may resolve the trade-off between permeability and selectivity, which is ubiquitous in NF membranes. The progress in interlayer technology has enabled the precise control of the interfacial polymerization (IP) process, which regulates the structure and performance of TFC NF membranes, resulting in a thin, dense, and defect-free PA selective layer. This review presents a summary of the latest developments in TFC NF membranes based on various interlayer materials. By drawing from existing literature, the structure and performance of new TFC NF membranes using different interlayer materials, such as organic interlayers (polyphenols, ion polymers, polymer organic acids, and other organic materials) and nanomaterial interlayers (nanoparticles, one-dimensional nanomaterials, and two-dimensional nanomaterials), are systematically reviewed and compared. Additionally, this paper proposes the perspectives of interlayer-based TFC NF membranes and the efforts required in the future. This review provides a comprehensive understanding and valuable guidance for the rational design of advanced NF membranes mediated by interlayers for seawater desalination and water purification.

## 1. Introduction

Nanofiltration (NF) membranes are a type of pressure-driven membrane that has the ability to intercept low-molecular-weight organic molecules and multivalent ions. These membranes have found widespread application in seawater desalination, drinking water purification, and wastewater treatment. The most commonly used commercial NF membranes are polyamide (PA) thin-film composite (TFC) membranes prepared by interfacial polymerization (IP), owing to their ease of processing, mature technology, and low cost. However, the trade-off between permeability and selectivity of TFC NF membranes has hindered the improvement of water permeability and solute retention rate simultaneously [1,2,3,4]. As a result, larger membrane areas and higher operating pressures are required for practical applications, which increases construction and operation costs. Hence, developing NF membranes with both high permeability and high retention is crucial for cost reduction.

PA TFC membranes have undergone tremendous development as the most successful commercialized product since the introduction of the concept of interfacial polymerization (IP) by Morgan in the early 1980s [5]. Despite the passage of nearly four decades, IP remains the foremost industrial technology for producing commercialized membranes. The typical IP process entails impregnating porous polymer support with a diamine aqueous solution, followed by exposure to a trimesoyl chloride organic solution. As the solubility of trimesoyl chloride in water is negligible, and the solubility of diamine is moderate in organic solvents [6], the polymerization reaction mainly occurs at the organic phase at the water-oil interface, where the diamine monomer diffuses from the water phase into the organic phase and rapidly reacts with the trimesoyl chloride. The resulting Janus reaction zone leads to the formation of an asymmetric PA nanofilm (with a total thickness of approximately 100 nm), where the amine and carboxyl groups are enriched on the water-phase and organic-phase sides, respectively. This gives rise to nanoscale heterogeneity in the PA nanofilm, which has been extensively discussed in studies by Freger et al. [7,8]. They concluded that the PA nanofilm features a negatively charged top layer, followed by a more densely packed positively charged sublayer.

PA TFC NF membranes mainly consist of a nonwoven fabric, a support layer, and a PA separation layer. The ultra-thin PA separation layer plays a crucial role in determining the flux and separation performance of the NF membrane. An ideal NF membrane should exhibit excellent water permeability and high removal rates of organic matter, multivalent salts, and other pollutants [9]. The thickness and crosslinking degree of the PA separation layer influence the overall separation performance of the TFC membrane. The traditional PA separation layer is approximately 100 nm, resulting in significant water transport resistance [10]. Therefore, it is necessary to reduce the thickness of the PA separation layer and increase the crosslinking degree to prepare high-performance NF membranes [11].

According to the classical theory of membrane separation and mass transfer, the permeability of a membrane is negatively correlated with the thickness of its selective layer [12]. Therefore, the thinner the selective layer, the higher the permeability [13]. The ultra-thinning of composite NF membranes is, in fact, the ultra-thinning of the PA separation layer. TFC NF membranes prepared by the IP method belong to diffusion-controlled reactions because of the rapid reaction rate of IP and the significant influence of the diffusion process of the water-phase monomer to the oil phase on the separation layer structure. Freger [7] summarized the kinetic model of the formation of the PA separation layer, and the thickness (δ) calculation formula is as follows:

(1) $$\delta \;\approx \;{\left[\frac{\mathrm{LD}}{\kappa \left(C_{a}f_{a}{+C}_{b}f_{b}\right)}\right]}^{1/3}$$

where L is the interface diffusion boundary layer thickness, D is the diffusion rate of the amine monomer to the water-oil interface, κ is the reaction rate constant between the two monomers, C_a_ is the local concentration of amine monomer, f_a_ is the functional-coefficient of the amine monomer, C_b_ is the local concentration of acyl chloride monomer, and f_b_ is the functional-coefficient of the acyl chloride monomer. By reducing the diffusion rate of amine monomers to the water-oil interface and increasing the local concentration of amine monomers, the thickness of the PA separation layer can be reduced.

To date, studies have shown that the physical and chemical properties of porous substrates significantly affect the formation of PA separation layers, presenting two challenges for the creation of ultra-thin and dense skin layers [11,14,15]. The first challenge is that existing porous substrates typically exhibit poor hydrophilicity and low porosity, leading to insufficient penetration of amine monomers on the substrate and resulting in defects in the formation of PA separation layers [16]. The second challenge is that the IP rate constant generated by the reaction of PIP and TMC is greater than 10^4^ L mol^−1^ s^−1^, making it difficult to kinetically control the IP process, which is not conducive to the formation of thin and dense PA separation layers [7,17].

In recent years, it has been demonstrated that the incorporation of an interlayer prior to the IP process on porous substrates is an effective approach for modifying the substrate [18,19]. The interlayer introduction induces a gutter effect, which enhances the transport efficiency of TFC NF membranes, leading to an increase in the membrane flux [20,21]. Furthermore, the interlayer can act as a reservoir for amine monomers, thereby elevating their concentration and regulating their release. The reaction between the amine and acyl chloride monomers on the interlayer generates an ultra-thin, defect-free, and dense PA active layer, which results in simultaneous enhancements of both the water permeability and salt rejection rate of the membrane [16,22]. This technology was first pioneered by Livingston et al. using a new type of composite substrate comprising a porous hydrophilic Cd(OH)_2_ nano strands sacrificial layer and the original UF substrate combined together, which has superior amine monomer storage capacity and controls the release of amine monomers to a certain extent. On the composite substrate, a 10 nm thick defect-free PA skin layer was formed by IP, which significantly improved solvent permeability while maintaining excellent salt retention capability. Since this groundbreaking work, numerous studies have focused on various interlayer materials, such as organic coatings and nanomaterials, to modify porous substrates and prepare TFC NF membranes with high permeability and high salt rejection properties. The TFC NF membranes with interlayers are referred to as TFCi in this article, while TFC0 refers to TFC NF membranes without interlayers.

Although the interlayer structure plays a crucial role, there is still a lack of a review of the influence of the interlayer structure on the performance of TFC NF membranes from a materials perspective. This article reviews the knowledge of TFC NF membranes based on various interlayer materials and provides useful information for researchers involved in developing high-performance TFC NF membranes.

## 2. Organic Interlayers

Organic hydrophilic polymers have abundant functional groups [23,24], which can easily be used to construct a uniform and continuous interlayer on a porous substrate. The interaction between the organic interlayer and the reactive monomer or the porous substrate can not only regulate the structure and performance of TFC PA NF membranes but also enhance the stability between the PA layer and the porous substrate [19,25,26,27]. So far, organic polymers used to construct interlayers mainly include polyphenols, ionic polymers, polymeric organic acid, and other types of organic matter.

### 2.1. Polyphenols

Polyphenols are natural compounds synthesized by plants that possess chemical characteristics similar to phenolic substances and exhibit strong antioxidant properties [28]. Polyphenolic chemistry for surface modification has gained significant attention in membrane science due to its simplicity and cost-effectiveness [29]. Polyphenols can also serve as an interlayer deposited on the support layer to enhance its hydrophilicity, which is beneficial in the manufacture of NF membranes [19,30].

Tannic acid (TA), a type of plant polyphenol, is widely used due to its rich terminal hydroxyl groups, including catechol and caramel alcohol structures, and broad sources [31]. Yang et al. [32] fabricated TFC PA NF membranes on TA-Fe nano-scaffolds. The TA-Fe nanomaterials exhibited improved absorption of amine monomers and served as a platform for the controlled release of the absorbed molecules. Additionally, the presence of a TA-Fe interlayer in the substrate resulted in smaller surface pores, which prevented the penetration of PA into the pores of the original substrate. The resulting TFCi membrane has a water permeability of 19.6 ± 0.5 L m^2−^ h^−1^ bar^−1^, which is one order of magnitude higher than that of the control TFC membrane (2.2 ± 0.3 L m^2−^ h^−1^ bar^−1^). At the same time, it increased the rejection of salts and the selectivity of divalent to monovalent ions (e.g., NaCl/MgSO_4_). However, the coordination bonds formed by TA-Fe are unstable in acidic environments, which limits its application. Zhao et al. [31] aimed to enhance the stability of the interlayer and achieved this by conducting a rapid in-situ coupling reaction between TA and diazonium salts (DDS), resulting in the construction of a stable diazo-based interlayer on a polysulfone (PSF) UF substrate. Compared to traditional NF membranes, the optimized new NF membrane’s water permeability was improved by 2.71 times.

In addition to TA, polyphenol-polyethyleneimine (PEI) coatings have also been used as interlayer materials. PEI possesses numerous primary amine groups, which can impart a significant positive charge to the interlayer, thereby increasing the surface potential of the NF membrane [33]. Additionally, PEI can form uniform nanoscale aggregates with polyphenols, which enhances interlayer stability and hydrophilicity [25,33]. For example, Zhai et al. [25] deposited large cyclic polyphenol molecules Noria-PEI onto a PSF UF substrate, where they underwent a Schiff base reaction to form covalent bonds and obtain a stable and uniform interlayer composed of a homogeneous nanoscale adhesive aggregate. The resulting Noria-PEI layer, positively charged, and the negatively charged PA separation layer endow the NF membrane with excellent rejection capabilities against divalent cations and anions. The optimized membrane exhibits a permeated flux of approximately 28 L m^2−^ h^−1^ bar^−1^ and rejection rates exceeding 96.0% for Na_2_SO_4_, MgSO_4_, MgCl_2_, and CaCl_2_. Inspired by this study, 5,5′,6,6′-tetrahydroxy-3,3,3′,3′-tetramethyl-1,1′-spirobisindane(TTSBI)-PEI [34] and catechol-PEI [33] have also been employed as interlayers to fabricate NF membranes with exceptional properties.

In addition to its co-deposition with PEI to form an interlayer, catechol can also be employed in combination with sodium periodate (SP) to prepare an interlayer. In a recent study, Tian et al. [35] demonstrated the preparation of an environmentally friendly polycarboxylic interlayer by mixing catechol and SP. Catechol, a polyphenolic compound, is susceptible to oxidation to form active o-quinones. Under the initiation of SP, these o-quinones can undergo phenolic polymerization and covalently polymerize with carboxyl groups. The introduction of carboxyl groups makes the interlayer hydrophilic, which can store more PIP molecules and provide a more uniform platform for IP, facilitating the formation of a thin and dense PA active layer. In comparison with traditional membranes, the modified TFC membrane has a 1.7-fold increase in permeability while maintaining a high rejection rate for Na_2_SO_4_ (98.42 ± 1.2%).

The interlayer of polyphenol has been extensively studied, yet the precise function and corresponding mechanisms leading to enhanced separation performance in TFCi NF membranes remain largely unknown. Yang et al. [20] developed a TFCi PA NF membrane with a polydopamine (PDA) interlayer. The optimal TFCi membrane flux was nearly an order of magnitude higher than that of the control TFC NF membrane. Additionally, the TFCi membrane exhibited efficient retention of inorganic salts and small organic molecules. Detailed mechanistic studies revealed that the membrane’s separation performance was enhanced through both the direct “gutter” effect of the PDA interlayer and its indirect influence on the PA layer, with the “gutter” effect exerting a more dominant role.

### 2.2. Ionic Polymers

In recent years, ion-polymer has also been used as an interlayer. The class of ionic polymers includes anionic polymers, cationic polymers, and zwitterionic polymers.

Hu et al. [36] introduced anionic polymer Poly(sodium 4-styrenesulfonate) (PSS) and a metal ion (Ca^2+^) complex interlayer on a PSF UF substrate to prepare a high-performance TFC NF membrane. The PSS-Ca^2+^ interlayer served as a nano scaffold, creating a more uniform IP platform that facilitated the formation of thinner and defect-free PA layers. As a macromolecular additive, PSS induced a diffusion-driven instability of the IP process, leading to the formation of a wrinkled PA layer with an increased surface area. This results in TFCi membranes that have four times the permeability (up to 22.15 ± 1.14 L m^−2^ h^−1^ bar^−1^) compared to traditional TFC0 membranes while still maintaining competitive rejection of Na_2_SO_4_. Deng et al. [37] used self-assembly to deposit anionic polymer sulfonated polyetherketone with cardo groups (SPEK-C) onto positively charged cadmium hydroxide (Cd(OH)_2_) nano strand, creating a highly permeable SPEK-C mesoporous interlayer. This interlayer not only enables the storage of a large number of water monomers but also generates electrostatic interactions with them, thereby temporarily slowing down their diffusion into the organic phase. The resulting PA NF membrane demonstrates excellent permeability and long-term operational stability.

In a recent study, Zhu et al. [38] incorporated a cationic polymer, quaternized crosslinked microgels (PNI6), onto a negatively charged hydrolyzed polyacrylonitrile (PAN) UF substrate to fabricate a TFC PA NF membrane. By introducing positively charged PNI6 microgels as the interlayer, the hydrophilicity of the product is improved, and the NF membrane surface becomes positively charged. Song et al. [39] provide another comparable instance where they have incorporated a cationic polymer, namely poly(allylamine hydrochloride) (PAH), onto a PSF UF substrate to fabricate an NF membrane with exceptional properties. The self-assembled network of strongly charged and highly hydrophilic ion aggregates exhibits a strong attraction to water amine monomers and temporarily disrupts their diffusion by applying a heterogeneous energy barrier to the organic phase, which then copolymerizes with the acyl chloride monomer. This instability in the diffusion driving force triggers the formation of internal voids on the back of the PA NF membrane.

**Figure 1:**
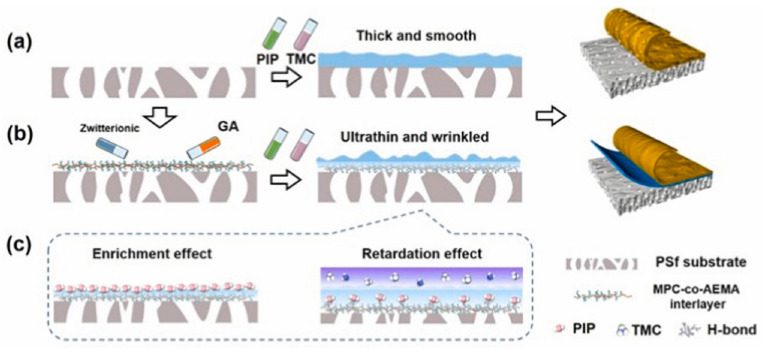
Schematic diagram of (**a**) conventional substrate and (**b**) P[MPC-co-AEMA] inter-mediated IP with the underlying mechanism of (**c**) enrichment effect and retardation effect. Reprinted with permission from ref. [40]. Copyright 2022 Elsevier.

In addition to anionic and cationic polymers, zwitterionic polymers have also been used in the preparation of NF membranes as interlayers. Song et al. [40] utilized a zwitterionic polymer, P[MPC-co-AEMA], consisting of 2-methacryloyloxyethyl phosphorylcholine (MPC) and 2-amino-ethyl methacrylate hydrochloride (AEMA), as an interlayer for the fabrication of a TFC NF membrane (Figure 1). The resulting membrane has an ultra-high water permeability of 20.4 L m^2−^ h^−1^ bar^−1^ (almost three times higher than the original PA membrane) and enhances the rejection of inorganic salts (the rejection rate for Na_2_SO_4_ is 96.8%). This was attributed to the abundant amine monomers in the P[MPC-co-AEMA] interlayer, which induced diffusion-driven instability, resulting in membrane sealing and inhibition of its growth. As a result, an ultra-thin PA NF membrane with a thickness of 12 nm, enhanced surface area, and enhanced crosslinking with a ridged surface structure was formed.

The data in Table 1 show that anionic polymers are the most effective interlayers for preparing TFC NF membranes, followed by zwitterionic polymers, and finally cationic polymers. This could be attributed to the fact that anionic polymers as interlayers can adsorb more quinine solution. According to Freger’s kinetics [7], an increase in amine monomer concentration is beneficial for producing thinner membranes and improving membrane flux. Additionally, anionic polymers as interlayers facilitate the preparation of membranes with a stronger negative charge, which is beneficial for intercepting Na_2_SO_4_. The adsorption capacity of zwitterionic and cationic polymers for PIP decreases in sequence, resulting in a corresponding decrease in the separation performance of TFC NF membranes prepared using these polymers.

### 2.3. Polymeric Organic Acid

Polymeric organic acids, due to their abundant carboxyl and hydroxyl groups, exhibit good hydrophilicity and are also used as interlayers. For example, Liu et al. [41] utilized hyaluronic acid (HA) as an interlayer material to fabricate a novel type of TFC NF membrane. The hydrogen bonds formed between HA and amine monomers act to inhibit the mass transfer of PIP, leading to the construction of a PA layer with enhanced electronegativity, reduced thickness, a wrinkled structure, and increased cross-linking degree. The resulting TFC NF membrane exhibited exceptional water permeability (29.53 L m^2−^ h^−1^ bar^−1^) and achieved high removal rates for Na_2_SO_4_ (94.90%) and perfluorohexane sulfonic acid (93.4%). Wang et al. [42] conducted a comparable investigation whereby they deposited poly(caffeic acid) (PCA) onto the surface of PAN UF membranes, constructing a composite interlayer and ultimately fabricating high-performance TFC NF membranes.

### 2.4. Other Organic Interlayers

**Figure 2:**
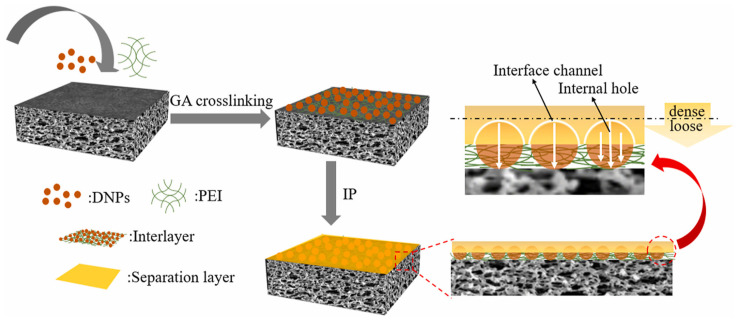
Process flowchart for the preparation of TFC NF membrane with an interlayer. Reprinted with permission from ref. [45]. Copyright 2022 Elsevier.

Researchers also used other types of organic materials to study the interlayer. For example, Zhu et al. [27] incorporated polyvinyl alcohol (PVA) as an interlayer on a PES UF substrate to fabricate TFC NF membranes. The interlayer provided a large surface area, high hydrophilicity, and high porosity, resulting in an NF membrane with a thickness of only 9.6 nm. The resulting membrane achieved an ultra-high water permeability of 31.4 L m^2−^ h^−1^ bar^−1^ and a high Na_2_SO_4_ rejection rate of 99.4%. In addition, the membrane showed good stability and scalability in pilot studies. Lan et al. [43] fabricated high-performance TFC NF membranes by constructing a hydrophilic and uniform gelatin (GT) interlayer on a PSF substrate. This interlayer not only influenced the diffusion rate of PIP to form a thin PA layer but also provided additional channels for water diffusion, significantly enhancing the permeability of the NF membrane. The permeability of the TFCi NF membrane was approximately 2.5 times higher than that of the control membrane while also achieving a high Na_2_SO_4_ removal rate. Liu et al. [44] prepared a novel TFC NF membrane with a poly (amidoxime) (PAO) organic interlayer. The PAO interlayer significantly enhanced the surface hydrophilicity of the PES substrate, and PAO could bind with PIP through hydrogen bonding and Lewis acid-base interactions, reducing the diffusion rate of PIP and causing instability at the oil-water interface during the IP process. This led to the formation of NF membranes with regular nanorod-like Turing structures and a thin PA layer thickness of only 18.2 nm. The optimized TFC NF membrane showed a permeating flux of up to 25.2 L m^2−^ h^−1^ bar^−1^ and a rejection rate of over 99% for Na_2_SO_4_. Chen et al. [45] developed a novel interfacial coating interlayer on a PSF UF substrate using glutaraldehyde (GA) cross-linked PEI and dextran nanoparticles (DNPs) (Figure 2). DNPs were semi-encapsulated within the PEI coating, while the exposed nanoparticles were embedded in the PA layer to form an interfacial channel during IP. The pure water permeation rate of the interlayer TFC NF membrane was 31.33 L m^2−^ h^−1^ bar^−1^, about four times that of the original membrane. Additionally, the NF membrane showed a significant improvement in the retention rate of various divalent salts, with the Na_2_SO_4_ retention rate exceeding 99%.

Through the comprehensive analysis of structural parameters and filtration performance of TFC NF membranes with organic material-interlayer (Table 1), comparative studies were conducted. Results showed that the polyphenol interlayer had been extensively studied in the development of novel TFC NF membranes compared to interlayers such as ionic polymers and polymeric organic acid. In order to improve the stability of TFC NF membranes, crosslinking agents such as PEI and GA are commonly employed to react with other organic materials to form the interlayer, while metal ions are used to chelate with other organic materials to create intermediate layers. Moreover, various porous substrates, including PES, PSF, and PAN, have been utilized in TFC NF membranes with organic interlayers, indicating the efficacy of the organic interlayer regardless of the substrate. As shown in Table 1, there is no clear correlation between monomer concentration and PA thickness, highlighting the significance of the organic interlayer in forming an ideal thin PA layer. After the incorporation of the organic interlayer, the membrane flux range of all TFCi NF membranes was between 10.1–31.4 L m^2−^ h^−1^ bar^−1^, and the retention rate range for Na_2_SO_4_ was between 93.4% and 99.4%. The Na_2_SO_4_ rejection performance of organic interlayer TFC NF membranes is equivalent to that of commercial NF membranes. Although the flux of these membranes is considerably higher than that of typical commercial NF membranes, further improvement is challenging. This is possibly due to the fact that while organic polymers can increase membrane flux by reducing the thickness of the PA layer and increasing its wrinkled structure, they may also block substrate pores and increase membrane resistance, ultimately hindering further flux improvement.

## 3. Nanomaterial Interlayer

### 3.1. Nanoparticles

Nanoparticles (NPs) are tiny particles ranging in size from 1 to 100 nanometers. They can be divided into three main types based on their composition: inorganic NPs, organic NPs, and organic-inorganic hybrid NPs.

#### 3.1.1. Inorganic NPs

**Figure 3:**
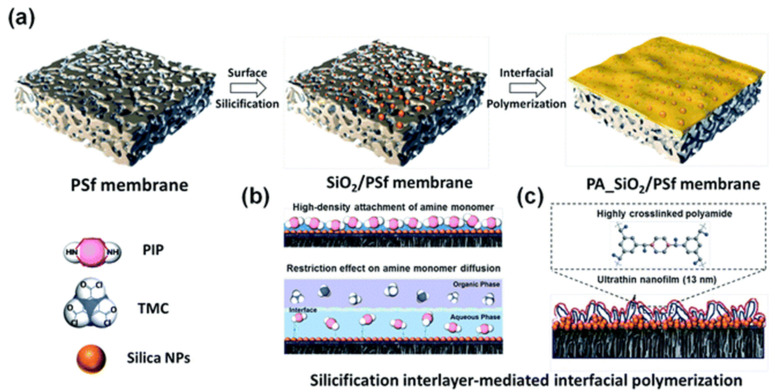
Schematic diagram of (**a**) silicification inter-mediated IP with the underlying mechanism of (**b**) amine monomer adsorption enrichment and diffusion restriction toward the organic phase for (**c**) developing ultra-thin, highly crosslinked PA nanofiltration membrane. Reprinted with permission from ref. [46]. Copyright 2022 Royal Society of Chemistry.

SiO_2_ is an inorganic nanoparticle material that exhibits notable hydrophilicity and chemical stability, making it a suitable interlayer material with good interfacial affinity and chemical reactivity. In order to enhance the stability of SiO_2_ on a PSF substrate, Song et al. [46] have employed an in-situ method to fabricate a siliconization interlayer (Figure 3). This process involves the hydrolysis of the silicate ester precursor in an aqueous environment to generate silanol, which subsequently reacts with the chloride acyl group located on the surface of the PSF membrane, forming a uniform siliconization layer. This technique can circumvent the issue of nanoparticle agglomeration and sedimentation during subsequent processing, thereby improving the stability of SiO_2_. The above-mentioned method only improves the stability of SiO_2_ on the substrate and does not mention the stability between SiO_2_ and the PA layer. To enhance the stability of SiO_2_ between the substrate and the PA layer concurrently, Wang et al. [47] incorporated PDA into SiO_2_. PDA can tightly bind to the SiO_2_ microsphere surface via a self-polymerization reaction, leading to the formation of a stable PDA@SiO_2_ interlayer between the PA layer and the poly(*m*-phenylene isophthalamide) (PMIA) substrate. This interlayer can augment the adhesion and compatibility between the PA layer and the PMIA substrate, consequently elevating the stability and mechanical strength of the composite NF membrane. Furthermore, PDA can create a cross-linking structure with PA molecules through hydrogen bonding, which further enhances the stability of the PA layer. The optimal membrane exhibits exceptionally high permeability, boasting a pure water flux of 31.37 ± 1.06 L m^2−^ h^−1^ bar^−1^, which is three times higher than that of traditional PA NF membranes, and a Na_2_SO_4_ rejection rate of up to 97.0%.

#### 3.1.2. Organic NPs

Covalent organic framework (COF), a type of organic nanoparticle material, possesses exceptional hydrophilicity and polymer affinity, along with ordered pores and adjustable pore size, which provide molecular sieving channels suitable for interlayers in NF membrane preparation. Han et al. [48] synthesized a COF (TPB-DMTP-COF) with uniform pore size for the NF membrane, serving as an interlayer material to regulate the physicochemical properties of the PA membrane. COFs, displaying affinity and hydrophilicity, can not only prevent non-selective interface voids but also decrease the diffusion rate of PIP towards the water/n-hexane interface by means of hydrogen bonding. The water permeability of the resulting TFCi membrane is significantly enhanced, nearly four times that of the unmodified PA membrane.

#### 3.1.3. Organic-Inorganic NPs

The metal-organic framework (MOF) is an organic-inorganic nanoparticle material composed of metal ions/clusters and organic scaffolds with highly controllable pore size and surface area, as well as an adjustable pore structure. These characteristics enable it to accelerate solvent transport by separating polymer chains or through its inherent pore structure. Additionally, the presence of organic ligands in MOFs improves their compatibility with the PA layer, reducing defect formation and enhancing interface stability. These features make MOFs a good interlayer material. Zhao et al. [49] introduced a zeolitic imidazolate framework-8 (ZIF-8) interlayer between a PA layer and a microporous poly(ether sulfone) (PES) membrane. Adding PSS to a ZIF-8 water solution containing Zn^2+^ can stabilize the ZIF-8 layer at the water/n-hexane interface and facilitate the uniform loading of ZIF-8 onto the substrate, effectively covering large pores. The surface of the PES membrane loaded with ZIF-8 becomes smoother and negatively charged, providing better conditions for IP to form a thin, loose, and defect-free TFC NF membrane. Additionally, the pure water permeability of the TFC membrane, containing a modified ZIF-8 interlayer, is twice that of the original TFC membrane.

### 3.2. One-Dimensional Nanomaterials

One-dimensional (1D) nanomaterials have the advantages of high porosity between materials, uniform pore size distribution, good hydrophilicity, and large surface charge, which can reduce the thickness of the PA layer, promote wrinkling morphology, and improve separation performance [16,50]. Therefore, 1D nanomaterials are suitable as interlayer materials for preparing NF membranes. The 1D nanomaterials are mainly divided into inorganic 1D nanomaterials and organic 1D nanomaterials.

#### 3.2.1. Inorganic 1D Nanomaterials

In recent studies, several researchers have utilized inorganic 1D nanomaterials as interlayers to prepare highly efficient NF membranes. Karan et al. [14] achieved a breakthrough in the manufacture of ultrathin films by conducting IP on Cd(OH)_2_ nanowire layers. The resulting nanowire interlayer facilitated the storage of diamine solution and control of diamine monomer release, ultimately leading to the formation of an ultrathin, defect-free PA skin layer. However, the subsequent removal of Cd(OH)_2_ nanowires may compromise the composite strength between the PA layer and substrate surface, leading to increased complexity in the manufacturing process. From a practical standpoint, interlayers exhibiting chemical stability are more desirable.

Carbon nanotubes (CNTs) that have been functionalized with carboxylic acid groups can serve as a robust mechanical support and facilitate IP by absorbing and storing aqueous amino solutions, making them suitable as interlayer materials [16,21,51,52]. Nonetheless, the presence of weak interfacial interactions between CNTs and porous substrates can result in deleterious delamination during practical applications [16,29,53]. In order to enhance the stability between the carbon nanotube interlayer and porous substrate, various methods have been developed, such as coating CNTs with PDA, carboxylating multi-walled carbon nanotubes (MWCNTs), employing brush painting to disperse single-walled carbon nanotubes (SWCNTs), and using inkjet printing technology to prepare stable CNT interlayers. For example, Zhu et al. [54] developed a PDA-coated SWCNT (PDA/SWCNT) ultra-thin film as a support layer for the preparation of TFC NF membranes. The PDA/SWCNTs composite material has a high porosity, smooth surface, and excellent hydrophilic properties. PDA forms strong interaction forces between the substrate and the PA layer, increasing adhesion and improving the stability of the composite material. The resulting TFCi NF membrane showed exceptional performance with a permeate flux of 32 L m^2−^ h^−1^ bar^−1^ and a 95.9% rejection rate for divalent ions. Wu et al. [16] conducted carboxylation of MWCNTs and utilized them as interlayers on the MF substrate to fabricate TFC NF membranes. The introduction of carboxylated MWCNTs can enhance the compatibility and adhesion between the interlayer and the substrate, leading to improved stability and performance of the TFC NF membrane. Gao et al. [51] employed a brush painting to disperse SWCNTs onto a polymer MF support, creating a network of SWCNTs as an interlayer. After subjecting the interlayers to soaking experiments for 30 days, all interlayers remained intact, and there was no peeling of SWCNT films from the underlying PES MF substrate. This confirms that brush painting is capable of producing interlayers with excellent chemical and mechanical stability. This approach enabled the formation of a 15-nm-thick PA layer, and the resulting TFCi NF exhibited an extremely high water permeation rate (~40 L m^2−^ h^−1^ bar^−1^) and a rejection rate of 96.5% for Na_2_SO_4_. Park et al. [52] used inkjet printing technology to prepare stable SWCNT interlayers, which is because inkjet printing technology can achieve high precision, high speed, and reproducibility of deposition, thereby allowing the morphology and distribution of materials to be controlled at the nanoscale.

CNTs have been used as an interlayer to prepare a thin PA active layer with a thickness of nearly 10 nm, resulting in a membrane with higher permeability. Although this strategy of reducing the active layer is effective in improving membrane permeability, further improving membrane performance under this strategy is limited because preparing thinner defect-free active layers than the state-of-the-art will become very challenging. Without losing other features, increasing the effective permeable area of the ultra-thin PA active layer is a preferred approach. Wang et al. [55] created a PA active layer with a widely crumpled surface morphology by loading ZIF-8 NPs onto SWCNTs/PES scaffolds, with ZIF-8 as a sacrificial template, thereby creating a higher specific surface area and larger proportion of highly permeable PA skin layers. The resulting membrane exhibits high permeability of up to 53.5 L m^2−^ h^−1^ bar^−1^ and a rejection rate of over 95% for Na_2_SO_4_.

According to Table 2, it can be observed that existing studies mainly use SWCNTs as the interlayer in the preparation of TFCi NF membranes, which show better performance compared to TFCi NF membranes prepared with MWCNTs as the interlayer. This is likely due to the smaller diameter of SWCNTs, which is advantageous for constructing a high-porosity interlayer that can store more PIP solution. According to Freger kinetics [7], an increase in the concentration of amine monomers is favorable for producing thinner membranes and improving membrane flux.

However, further research is necessary to understand how changes in interlayer structure and properties affect the formation and transport properties of PA. Recent studies have demonstrated the potential of tailoring interlayer properties to enhance the NF performance of TFC membranes. For example, Gong et al. [56] investigated the interlayer’s role in regulating the structure and performance of PA by introducing an SWCNT interlayer on a PSF MF substrate to prepare a TFC NF membrane. Through manipulation of the properties of the CNT interlayer, such as its thickness and surface pore size, the structure and performance of the PA active layer can be carefully tailored to improve its NF performance. The optimized TFCi NF membrane displayed a high rejection rate for divalent salts and dyes, as well as a highly pure water flux of approximately 21 L m^2−^ h^−1^ bar^−1^. To explore the water transport mechanism in the TFCi NF membrane, Long et al. [21] analyzed an MWCNTs-incorporated TFCi membrane with an identical PA rejection layer but different interlayer thicknesses. It was observed that increasing the thickness of the MWCNTs interlayer improved the transport of water in the transverse direction, leading to an enhanced gutter effect, but also increased the hydraulic resistance in the normal direction. The optimal permeate flux was achieved at an interlayer thickness of 13.0 ± 0.7 L m^2−^ h^−1^ bar^−1^. In this study, it was demonstrated that the TFCi NF membrane reduced membrane fouling and improved the reversibility of fouling compared to the control membrane without an interlayer, which could be ascribed to its more uniform water flux distribution.

#### 3.2.2. Organic 1D Nanomaterials

**Figure 4:**
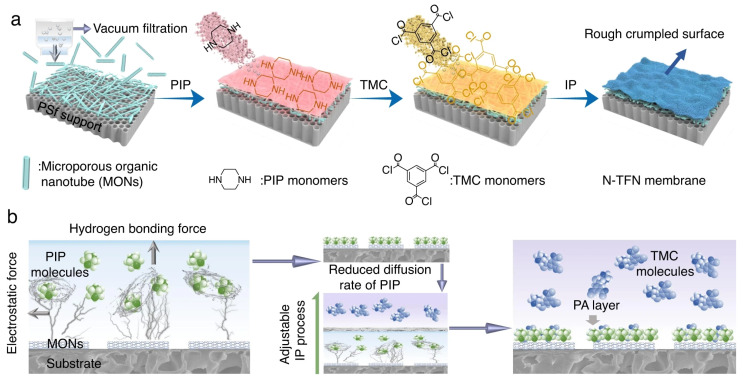
Schematic diagram of (**a**) MON inter-mediated IP with the underlying mechanism of (**b**) hydrogen bonding and electrostatic interactions. Reprinted with permission from ref. [63]. Copyright 2022 Springer.

Inorganic 1D nanomaterials, such as Cd(OH)_2_ [14] and CNTs [16,21,51,52,54,56], have been widely studied as interlayer materials for NF membranes, but their toxicity and poor compatibility with substrates and PA layers have been significant drawbacks [57,58]. To overcome the toxicity and compatibility issues associated with inorganic 1D nanomaterials, researchers have shifted their focus to developing hydrophilic, environmentally friendly, and non-toxic organic 1D nanomaterials as interlayer materials. Several recent studies have demonstrated the effectiveness of this approach, utilizing different organic nanomaterials as interlayers to achieve highly efficient NF membranes with impressive permeation rates and rejection capabilities. For example, Wang et al. [53] prepared NF membranes on a nanoporous substrate using a non-toxic and environmentally friendly cellulose nanocrystal interlayer. The high aspect ratio and hydrophilicity of the cellulose nanocrystals (CNCs) interlayer can store aqueous diamine monomers, slow down IP, and prepare thinner membranes. The constructed TFCi NF membrane exhibits an ultra-high permeation rate of up to 34 L m^2−^ h^−1^ bar^−1^ and a rejection rate for Na_2_SO_4_ exceeding 97%. Similarly, Wang et al. [59] used highly hydrophilic and high surface area cellulose nanofibers (CNFs) as an interlayer to prepare TFC NF membranes. Compared to the control membrane, the water flux of the TFC NF membrane with interlayer CNFs was significantly increased by 270%, and the MgCl_2_ rejection rate was as high as 96.8%. Ji et al. [60] prepared a PA NF membrane on a UF substrate using a polyaniline nanofibers interlayer. The acid doping chemical reaction of polyaniline allows for tunable pore size and surface charge in the PA layer, which enhances ion separation. The optimized TFCi NF membrane demonstrates an ultra-high water permeation rate of 49.7 L m^2−^ h^−1^ bar^−1^ and good retention for Na_2_SO_4_ and MgSO_4_, equivalent to 98.7% and 97.9% salt rejection, respectively. Similarly, Guo et al. [61] developed a sandwich-enhanced NF membrane using self-doped sulfonated polyaniline (SPANI) nanofibers as an interlayer. The degree of sulfonation in the interlayer was varied and increasing the sulfonation degree led to improved hydrophilicity and thinner, defect-free PA layers. The high sulfonation degree of SPANI also gives the TFCi membrane a high water permeation rate of up to 29.35 ± 1.23 L m^2−^ h^−1^ bar^−1^ while retaining good rejection capability for Na_2_SO_4_ (98.92 ± 0.45%) and MgSO_4_ (96.21 ± 0.75%). However, the effect of combining the polyaniline interlayer with other chemical substances on the separation performance of TFCi NF membranes is still uncertain. Guo et al. [62] further investigated the impact of different types of alkaline solutions combined with polyaniline on the separation performance of TFCi NF membranes. The addition of NaHCO_3_ to the SPANI interlayer facilitated the formation of a thin and defect-free polyamide (PA) selective layer with a wrinkled and nanobubble dual morphology. The optimized TFCi NF membrane demonstrated a remarkable permeate flux of 35.35 L m^−2^ h^−1^ bar^−1^ and exhibited excellent rejection capabilities towards Na_2_SO_4_ (98.95 ± 0.29%) and MgSO_4_ (95.37%). Finally, Han et al. [63] used high porosity microporous organic nanotubes (MONs) as an interlayer and prepared a 15-nanometer-thick PA layer through IP (Figure 4). Prior to the IP reaction, a high-porosity and interconnected MON layer was added onto the membrane, which can form a turing structure PA membrane, increase the microporosity rate, and reduce thickness. The optimized TFCi NF membrane achieved a significant water permeability of 41.7 L m^2−^ h^−1^ bar^−1^ under alkaline conditions (pH = 10) and high retention rates for boron (78.0%) and phosphorus (96.8%). Overall, the use of organic 1D nanomaterials as interlayers in NF membranes has shown great potential for improving their performance and efficiency, and these studies demonstrate the effectiveness of this approach in achieving highly efficient NF membranes for a variety of applications.

### 3.3. Two-Dimensional Nanomaterials

Two-dimensional (2D) nanomaterials possess high aspect ratios and significant lateral dimensions, rendering them conducive for covering large substrate pores with multiple stacked layers, thereby facilitating the attainment of a smooth and uniform surface that is indispensable for the IP process [64]. They can be divided into three main types based on their composition: inorganic 2D nanomaterials, organic 2D nanomaterials, and organic-inorganic 2D nanomaterials.

#### 3.3.1. Inorganic 2D Nanomaterials

In recent years, researchers have employed inorganic 2D nanomaterials, such as graphene oxide (GO), MXene, and MoS_2_ as interlayers to develop NF membranes. GO nanosheets can achieve ultrafast water transport through nanochannels constructed from pristine graphene [65] thanks to their excellent mass transfer properties, such as ultra-low friction and ultra-high capillary effect. Moreover, GO nanosheets possess various oxygen-containing functional groups on their surfaces and edges, making them highly promising for use as interlayers in the fabrication of NF membranes. Nonetheless, GO does not undergo a chemical reaction or strong interaction with most substrates, such as PSF and polyether sulfone (PES), thereby making it difficult to attain stable adhesion, resulting in interlayer delamination during the filtration process [66,67]. Crosslinking with Pebax^®^ 1657 and Toluene diisocyanate (TDI) can improve the stability of graphene oxide on PAN [68]. Inspired by this, to mitigate the instability of the GO interlayer, Zhang et al. [69] introduced PVA into GO, followed by crosslinking with GA. GA acts as a cross-linking agent, reacting with PVA to form a crosslinked structure, which fixes the GO nanosheets on the surface of the MF substrate. The resulting crosslinked structure enhances the stability and durability of the interlayer and improves the selectivity and permeability of the TFC NF membrane. Testing the anti-fouling performance of TFCi NF membrane using BSA-contaminated water samples as feed, it was found that TFCi NF membrane exhibited a lower flux decline compared to TFC0 NF membrane and had a higher flux recovery rate (FRR), indicating that TFCi NF membrane has superior anti-fouling performance. In addition, the stacking of GO nanosheets is also a barrier to its use as an interlayer because it increases the resistance of water passing through it. To reduce GO stacking, Wang et al. [70] inserted and crosslinked dispersed sub-5 nanometer silica NPs in situ in the GO interlayers. The hydrophilic NPs locally widened the interlayer channels, enhancing the permeability of solvents. This method has the potential to solve the aggregation problem of GO as an interlayer.

**Figure 5:**
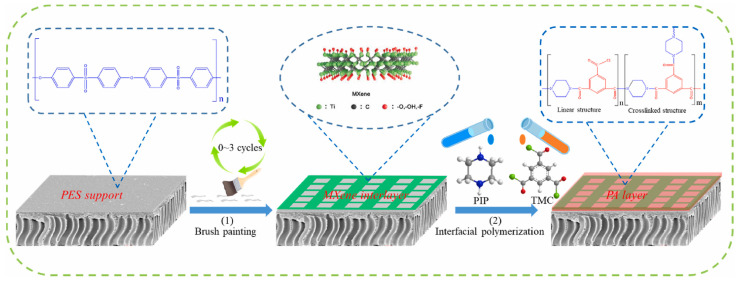
Schematic diagram of MXene inter-mediated IP. Reprinted with permission from ref. [75]. Copyright 2021 Elsevier.

MXene, an inorganic 2D nanomaterial produced by selective etching, has attracted significant attention due to its hydrophilic end groups and mechanical flexibility [71]. Xu et al. [72] have fabricated a high-performance TFC NF membrane by utilizing the 2D sheet-like interlayer of MXene for IP. The MXene layer facilitated the absorption of active monomers, and higher amine monomer concentrations promoted self-sealing and self-termination of IP, resulting in thinner PA films with suppressed formation inside the substrate pores. The resulting TFCi NF membrane exhibited over 96% rejection of Na_2_SO_4_ and an excellent permeability of 45.7 L m^2−^ h^−1^ bar^−1^, which was nearly 4.5 times that of the TFC0 NF membrane. However, the post-treatment process involving oxidants made the obtained high-performance PA NF membrane difficult to scale up, and the ultra-thin PA layer was transferred onto a PES support after dissolving the MXene layer, causing instability of PA membranes and deterioration of membrane performance [73,74]. In order to address this issue, Zhu et al. [75] prepared an efficient NF membrane by using MXene as an interlayer in an assisted IP process (Figure 5). The presence of the MXene layer increased the hydrophilicity of the original support and the adsorption of PIP, resulting in a faster IP reaction. The resulting MXene-interlayer NF membrane exhibited a rougher surface, higher hydrophilicity, higher electronegativity, and a denser skin layer. The MXene-interlayer NF membrane achieved an excellent water permeability of 27.8 ± 2.1 L m^2−^ h^−1^ bar^−1^ while also achieving satisfactory selectivity, overcoming the trade-off between water permeability and salt selectivity.

The aforementioned examples illustrate the successful use of MXene as an interlayer material for the preparation of high-flux TFC NF membranes. This is because the introduction of the interlayer generates a gutter effect [20], which reduces the total resistance of the TFC nanofiltration membrane (sum of resistance in substrate, interlayer, and PA layer) [76], resulting in an increase in membrane flux. When MXene 2D nanosheets are used as interlayers, the excessive stacking of MXene functional groups and water molecules via hydrogen bonding decreases the interlayer spacing and elevates the transport resistance of water through the MXene interlayer itself. According to the resistance-in-series model of water transport resistance [77], the interlayer of an ideal TFCi membrane itself should have low transport resistance. Therefore, to address this issue, some researchers have investigated the insertion of other nanomaterials between MXene interlayers to regulate the interlayer spacing of the 2D channels, thus reducing the resistance of the MXene interlayer itself. For instance, Wang et al. [78] employed stacked MXene nanosheets as the interlayer and embedded Fe_3_O_4_ NPs as a sacrificial template to adjust the interlayer spacing. However, foreign insertions are inherently unstable for the long-term operation of the membrane. Alternatively, Fu et al. [79] successfully grew TiO_2_ in situ on MXene nanosheets via PMS oxidation. The TiO_2_ NPs can firmly bind to the MXene layer and enlarge the interlayer spacing, thereby mitigating the additional transport resistance of water molecules. Furthermore, the MXene-TiO_2_ heterostructure material exhibits a higher specific surface area than MXene [80,81], which facilitates the collection of more amide monomers to form a crumpled PA selective layer. This method has successfully produced high-performance NF membranes.

Recently, MoS_2_ has garnered significant attention as a promising inorganic 2D nanomaterial for membrane separation due to its exceptional mechanical stability, robust interlayer forces, and simple synthesis process. Cao et al. [76] fabricated a high-performance TFC NF membrane on a PSF substrate coated with MoS_2_. The MoS_2_-interlayer TFC NF membrane demonstrated superior roughness and crosslinking compared to the control group TFC NF membrane, owing to the enhanced confinement effect of interfacial degassing and the improved adsorption of amine monomers by the MoS_2_- interlayer. Additionally, the PA layer thickness of the MoS_2_-interlayer TFC membrane, which ranged from 60 to 85 nm, could be finely tuned by altering the thickness of the MoS_2_ interlayer. The optimized TFCi NF membrane exhibited a remarkable rejection rate of 96.8% for Na_2_SO_4_ and a desirable permeability of approximately 15.9 L m^2−^ h^−1^ bar^−1^, which was approximately 2.4 times greater than that of the TFC0 NF membrane.

#### 3.3.2. Organic 2D Nanomaterials

In addition to inorganic 2D nanomaterials, organic 2D nanomaterials have also attracted the interest of researchers. COFs are a class of organic 2D nanomaterials known for their remarkable porosity and strong covalent bonding within the all-organic skeleton [82]. Most COFs exhibit a highly ordered honeycomb-like structure with tunable pore sizes and layered structures [83], which makes them well-suited for selective small molecule separation through size exclusion. The exceptional water stability, high adsorption capacity, and well-defined structure make COFs promising candidates for storing amine monomers, thus making them suitable as interlayer materials. For example, Wu et al. utilized [84] a PDA-COF interlayer to mediate IP, resulting in ultra-thin composite membranes with enhanced NF performance. The PDA-COF interlayer with specific surface hydrophilicity and high porosity was able to control the adsorption/diffusion of amine monomers, leading to the formation of an ultra-thin and dense PA layer that reduced the thickness from 79 nm to 11 nm. The optimized TFCi NF membrane showed excellent desalination rate and dye rejection with outstanding water permeability of 20.7 L m^2−^ h^−1^ bar^−1^. Additionally, the PDA-COF interlayer significantly enhanced the interfacial interaction between the PA layer and PAN substrate, providing the composite membrane with excellent structural stability. Another example is Yuan et al. [85] developed a composite substrate using COF nanosheets (CONs) deposited on an MF membrane through vacuum-assisted assembly, which allowed for the regulation of IP and produced a highly porous and super hydrophilic ultra-thin PA skin layer. The high porosity and superhydrophilicity of CONs in the composite substrate allowed for high storage capacity and uniform distribution of amine monomers, and by varying the loading amount of CONs, it was possible to regulate the monomer storage capacity, accelerate self-sealing and self-termination of IP, and produce ultra-thin PA skin layers ranging from 70 nm to below 10 nm. Moreover, due to the highly porous structure of CONs, the composite substrate exhibited almost no additional resistance to water transport. The optimized TFCi NF membrane exhibited outstanding water permeability of 53.55 L m^2−^ h^−1^ bar^−1^, with a rejection rate of 94.3% for Na_2_SO_4_. 

#### 3.3.3. Inorganic-Organic 2D Nanomaterials

In a recent study, inorganic-organic 2D nanomaterials were also applied to prepare interlayer materials. For example, Cheng et al. [86] have developed a TFC NF membrane by incorporating MOFs as an interlayer in a PSF-based substrate. Through controlling the thickness of the MOFs (copper-tetrakis(4-carboxyphenyl) porphyrin, Cu-TCPP) interlayer, the structural parameters, such as effective filtration area, selective layer thickness and pore size, as well as the physicochemical properties of the membrane could be precisely adjusted. The experimental results indicate that the MOFs nanosheets with appropriate deposition density as interlayers could effectively increase the crosslinking degree by adsorbing PIP monomers while simultaneously reducing the thickness of the PA layer, resulting in enhanced intrinsic permeability. The optimized TFCi NF membrane demonstrates exceptional water permeability of 32.7 L m^2−^ h^−1^ bar^−1^, as well as a high selectivity of 271.7 for NaCl/Na_2_SO_4_.

A thorough analysis was conducted to compare the structural parameters and filtration performance of TFC NF membranes with nano-material interlayers, as outlined in Table 2. The results demonstrate that 1D and 2D nanomaterial interlayers have been extensively studied for the development of new TFC NF membranes compared to nanoparticle interlayers. To enhance the stability of TFC NF membranes, PDA and nanomaterials are commonly combined. PDA is a bio-adhesive with a polyphenolic structure that interacts with various surfaces and has good biocompatibility and biodegradability. Therefore, the inclusion of PDA in NF membrane preparation improves the stability of TFC membranes. A variety of porous substrates such as PES, PSF, PMIA, PAN, Nylon, and PVDF have been used as substrates for nanomaterial interlayers. While both MF and UF substrates have been widely used for interlayer materials, NF membranes with ultrahigh permeate flux (>40 L m^2−^ h^−1^ bar^−1^) have been obtained using MF substrates, which may be attributed to the high permeability of the MF substrate itself. Table 2 highlights that there is no obvious correlation between monomer concentration and PA thickness, emphasizing the critical role of nanomaterial interlayers in creating an optimal thin PA layer. Following the addition of nanomaterial interlayers, the flux range of all TFCi NF membranes falls between 9.48–53.55 L m^2−^ h^−1^ bar^−1^, while the Na_2_SO_4_ retention range is between 91% and 99.9%. When compared to organic materials, nanomaterials exhibit higher permeability but equivalent salt rejection performance as interlayers. This may be due to the generally higher porosity of nanomaterials, which results in lower resistance as an interlayer in comparison to organic materials.

**Table 2:** Structure and performance of TFC NF membranes with nanomaterial interlayer.

Category	Nanomaterial Used	Porous Substrate	IP Condition (Optimum)	Polyamide Thickness (nm)	Water Flux (L m^−2^ h^−1^ bar^−1^)	Salt Rejection	Year [Ref]
NPs	ZIF-8-PSS	PES MF substrate (0.22 μm)	2 *w*/*v*% PIP/water, 0.13 *w*/*v*% TMC/n-hexane 1 min reaction	235	9.6	91% Na_2_SO_4_	2021 [49]
NPs	SiO_2_	PSF UF substrate (Mw 30,000)	0.2 *w*/*v*% PIP/water, 0.1 *w*/*v*% TMC/n-hexane 30 s reaction	15	14.5	98.7% Na_2_SO_4_	2021 [46]
NPs	COF	PSF UF substrate	0.1 wt% PIP/water, 0.1 wt% TMC/n-hexane 1 min reaction	44	35.7	98.9% Na_2_SO_4_	2022 [48]
NPs	PDA@SiO_2_	PMIA substrate	1 wt% PIP/water, 0.1 *w*/*v*% TMC/n-hexane 30 s reaction	20.9	31.37	97% Na_2_SO_4_	2022 [47]
1D	PDA/SWCNT (5–30 μm × Φ < 2 nm)	PES MF substrate (0.4 μm)	0.025 *w*/*v*% PIP/water, 0.02 *w*/*v*% TMC/n-hexane 30 s reaction	12	32	95.9% Na_2_SO_4_	2016 [54]
1D	MWCNTs (50 μm × Φ 8–15 nm)	PSF UF substrate (Mw 30,000)	0.15 *w*/*v*% PIP/water, 0.02 *w/v*% TMC/n-hexane 30 s reaction	NA	17.57	95% Na_2_SO_4_	2016 [16]
1D	Cellulose nanocrystal (0.2–0.5 μm × Φ 20–50 nm)	PES MF substrate (0.22 μm)	0.5 mg/mL PIP/water 0.5 mg/mL TMC/n hexane 2 min reaction	45	34	97.7% Na_2_SO_4_	2017 [53]
1D	PDA@ZIF-8/PDA@SWCNTs	PES MF substrate	2.5 mg/mL PIP/water 2 mg/mL TMC/n-hexane 30 s reaction	NA	53.5	95% Na_2_SO_4_	2018 [55]
1D	SWCNT (5~30 μm × Φ 1–2 nm)	PES MF substrate (0.45 μm)	0.125 *w*/*v*% PIP/water, 0.1 *w*/*v*% TMC/n-hexane 30 s reaction	15	40	96.5% Na_2_SO_4_	2019 [51]
1D	PDA/SWCNTs (5–30 μm × Φ < 2 nm)	PES MF substrate (0.22 μm)	0.05 wt% PIP/water, 0.02 wt% TMC/n-hexane 30 s reaction	29	21	98.5% Na_2_SO_4_	2019 [56]
1D	SWCNT (1~3 μm × Φ 1–2 nm)	PES MF substrate (0.22 μm)	0.15 wt% PIP/water, 0.1 wt% TMC/n-hexane 30 s reaction	42.6	18.24	97.88% Na_2_SO_4_	2021 [52]
1D	Polyaniline nanofibers	PES MF substrate (0.45 μm)	1 *w*/*v*% PIP/water, 0.15 *w*/*v*% TMC/n-hexane 30 s reaction	11	49.7	98.7% Na_2_SO_4_	2022 [60]
1D	sulfonated polyaniline nanofibers (0.58 μm × Φ 80 nm)	PES MF substrate (0.45 μm)	1 *w*/*v*% PIP/water, 0.15 *w*/*v*% TMC/n-hexane 30 s reaction	48	29.35	98.92% Na_2_SO_4_	2022 [61]
1D	Cellulose nanofibers	PSF UF (Mw 50,000)	0.3 *w*/*v*% PEI/water, 0.1 *w*/*v*% TMC/n-hexane 1min reaction	105.4	13.3	96.8%MgCl_2_	2022 [59]
1D	MWCNTs	PES UF substrate (Mw 20,000)	0.2 wt% PIP/water, 0.1 wt% TMC/n-hexane 2 min reaction	NA	13	NA	2022 [21]
1D	Microporous organic nanotubes	PSF substrate	0.1 wt% PIP/water, 0.1 wt% TMC/n-hexane 1 min reaction	15	41.7	98.7% Na_2_SO_4_	2022 [63]
1D	sulfonated polyaniline	PES MF substrate (0.45 μm)	1 wt% PIP/water, 0.15 wt% TMC/n-hexane 30 s reaction	43	35.35	98.95% Na_2_SO_4_	2023 [62]
2D	PDA-COF nanosheets (lateral size 60–130 nm)	PAN UF substrate (Mw100,000)	0.1 *w*/*v*% PIP/water, 0.1 *w*/*v*% TMC/n-hexane 2 min reaction	11	20.71	93.4% Na_2_SO_4_	2019 [84]
2D	COF nanosheets (lateral size 250 nm)	PES MF substrate (0.10 μm)	0.15 wt% PIP/water, 0.15 wt% TMC/n-hexane 2 min reaction	7	53.55	94.3% Na_2_SO_4_	2019 [85]
2D	GO nanosheets	Nylon MF substrate (0.22 μm)	0.5 *w*/*v*% PIP/water, 0.5 *w*/*v*% TMC/n-hexane 2 min reaction	NA	30.3	93.56% Na_2_SO_4_	2020 [87]
2D	MXene nanosheets	PES UF substrate	1 wt% PIP/water, 0.1 wt% TMC/n-hexane 30 s reaction	15	27.8	99.9% Na_2_SO_4_	2021 [75]
2D	MXene nanosheets	PES MF substrate (0.22 μm)	0.2 wt% PIP/water, 0.1 wt% TMC/n-hexane 30 s reaction	20	45.7	96% Na_2_SO_4_	2021 [72]
2D	GO@PVA/GA	PSF substrate	0.3 wt% PIP/water, 0.06 wt% TMC/n-hexane 30 s reaction	15	15.8	99.7% Na_2_SO_4_	2021 [69]
2D	MOFs nanosheets	PES UF substrate (Mw 20,000)	0.15 wt% PIP/water, 0.1 wt% TMC/n-hexane 1 min reaction	23.5	32.7	99.7% Na_2_SO_4_	2022 [86]
2D	MoS_2_ (average size of 2 μm)	PES UF substrate (Mw 150,000)	1 wt% PIP/water, 0.15 wt% TMC/n-hexane 1 min reaction	72.7	15.9	96.8% Na_2_SO_4_	2022 [76]
2D	TiO_2_ nanosheets (lateral size 0.6–1.2 μm)/TiO_2_ NPs	PVDF MF substrate (0.45 μm)	1 wt% PIP/water, 0.1 wt% TMC/n-hexane 15 s reaction	30	36.3	NA	2023 [88]
2D	MXene-TiO_2_	PSF UF substrate (Mw 20,000)	0.75 wt% PIP/water, 0.038 wt% TMC/n-hexane 2 min reaction	30.25	11.10	98.29% MgSO_4_	2023 [79]
2D	MXene Nanosheets/Fe_3_O_4_ NPs	PSF UF substrate (Mw 20,000)	0.75 wt% PIP/water, 0.038 wt% TMC/n-hexane 2 min reaction	22	9.48	>97% MgSO_4_	2023 [78]

## 4. Comparison of Performance of TFCi NF Membranes

### 4.1. Comparison of Performance of TFCi NF Membranes with TFC0 NF Membranes

**Figure 6:**
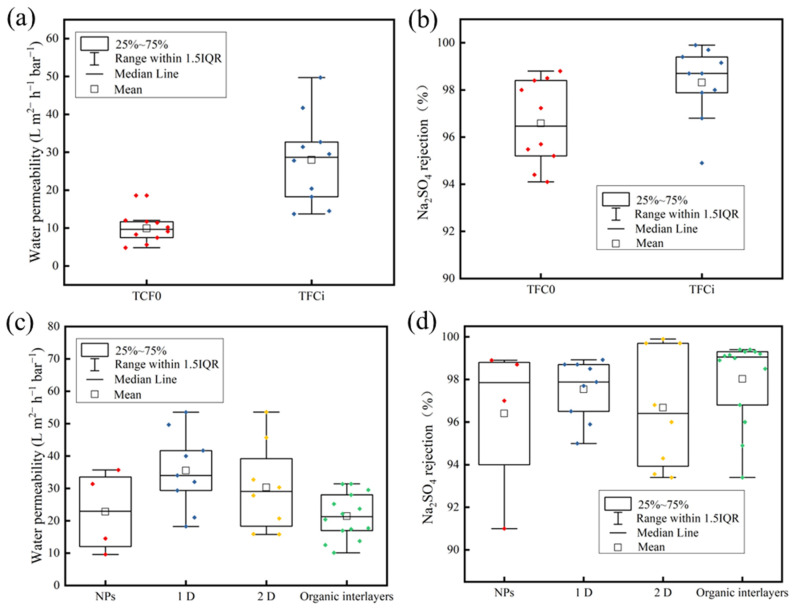
Statistical analysis of (**a**) water permeability and (**b**) Na_2_SO_4_ rejection of TFC0 and TFCi NF membranes [27,35,40,41,46,52,60,63,75,86]. Statistical analysis of (**c**) water permeability and (**d**) Na_2_SO_4_ rejection based on NPs [46,47,48,49] 1D [51,52,53,54,55,56,60,61,63], 2D nanomaterials [69,72,75,76,84,85,86,87], and Organic interlayers [25,27,31,34,35,36,38,39,40,41,42,43,44,45]. This is a rough comparison as the substrates used in these publications are not similar, and the substrate has an impact on the formation of PA membranes by IP. Moreover, the IP conditions are different, which also affects the separation performance of the membranes.

The present study provides a comprehensive review of the separation performance of interlayer TFC N membranes based on existing literature. The results indicate that the TFCi NF membrane exhibits significantly improved separation performance compared to the TFC0 NF membrane. Specifically, the average flux of the TFCi NF membrane increased from 9.92 L m^2−^ h^−1^ bar^−1^ to 27.968 L m^2−^ h^−1^ bar^−1^, representing an increase of 182%, as demonstrated in Figure 6a. Additionally, the average rejection rate of Na_2_SO_4_ for the TFCi NF membrane increased from 96.58% to 98.31%, as illustrated in Figure 6b, which suggests that the introduction of an interlayer has the potential to enhance flux without compromising the selectivity of the membrane. The observed improvements in separation performance can be attributed to the formation of a thin, dense, and crumpled TFC NF membrane after the incorporation of an interlayer. These findings demonstrate the promising potential of the interlayer strategy in overcoming the permeability-selectivity trade-offs of TFC membranes.

### 4.2. Comparison of Performance of TFCi NF Membranes Prepared Using Different Interlayer Materials

Figure 6 provides a comparison of the permeability and salt rejection rates of various materials. Specifically, Figure 6c demonstrates that the NF membrane incorporating a 1D nanomaterial as an interlayer exhibits the highest flux in terms of permeability. This can be attributed to the high aspect ratio, internal porosity, and uniform pore size distribution of 1D nanomaterials, which contribute to a reduction in PA layer thickness and the attainment of a crumpled morphology, ultimately resulting in higher permeability. Similarly, the NF membrane created with 2D nanomaterial as the interlayer also demonstrates a high flux due to the high surface area and unique physical and chemical properties of 2D nanomaterials, enabling them to establish a layered structure in two dimensions. This layered structure forms water channels that can be assembled into water channels by stacking nanosheets. However, 2D nanomaterials produce resistance to TFCi NF membranes, thus impeding membrane permeability improvement. By contrast, the organic interlayer exhibits the lowest flux because organic interlayers primarily consist of polymer materials with high molecular weight and large molecular size, leading to blockage of the NF membrane’s pore structure, thereby hampering the quantity and velocity of water molecules passing through the membrane. Additionally, the organic interlayer might form an extra resistance on the membrane’s surface and have adverse interactions with the active layer, reducing the NF membrane’s permeability. Figure 6d illustrates that the mean salt rejection rate of 2D materials is lower than that of 1D materials for sodium sulfate, which might be attributed to the fewer samples, the greater data fluctuations occur. Nevertheless, the maximum salt rejection rate of 2D materials surpasses that of 1D materials since 2D nanosheets inherently exhibit a blocking effect on salt. Finally, the NF membrane produced with an organic interlayer presents the highest mean salt rejection rate, potentially due to the abundant functional groups such as hydroxyl, carboxyl, and sulfonic acid groups in organic interlayers, which enhance membrane negative charge and facilitate the retention of negatively charged sodium sulfate.

## 5. Concluding Remarks and Future Perspectives

The incorporation of interlayers onto the substrate during the preparation of NF membranes has been demonstrated to effectively enhance their performance. Various materials, including NPs, organic polymers, 1D nanomaterials, and 2D nanomaterials, have been investigated as interlayers for TFC NF membrane preparation (Table 1 and Table 2). TFC NF membranes with high salt rejection and permeability have been developed using all types of interlayer materials. Among them, 1D nanomaterials exhibit better flux improvement, while organic and 2D nanomaterials demonstrate superior salt rejection ability. However, TFC NF membranes utilizing interlayers are still in the early stages of development. To advance the development of high-performance TFC NF membranes using interlayers, it is necessary to address several issues.

1. Interlayer materials. Considering that 1D nanomaterials are most effective in improving membrane flux, and organic polymers and 2D nanomaterials are good at improving salt rejection ability, composite materials such as organic/1D nanomaterials and 2D/1D nanomaterials may be candidate materials for preparing high-performance NF membranes. Some researchers have introduced PDA/CNT as an interlayer for the preparation of NF membranes [54,55,56]. In addition to enhancing the selectivity of the NF membrane, the function of PDA can also serve as an adhesive to encapsulate CNTs, thereby improving the stability of the TFC NF membrane. Some other researchers have directly used organic 1D nanomaterials (such as Polyaniline nanofibers [60] and microporous organic nanotubes [63]) as interlayers to prepare NF membranes with not only extremely high flux (>40 L m^2−^ h^−1^ bar^−1^) but also high salt rejection ability (rejection of Na_2_SO_4_ > 98%) (see Table 2). Organic 1D nanomaterials have more hydrophilic functional groups and better chemical stability than inorganic 1D nanomaterials (such as CNTs), combining the advantages of both inorganic 1D nanomaterials and organic polymers. Moreover, to date, there have been no reports on 2D/1D nanomaterials used as interlayer materials for preparing NF membranes, although 2D/1D nanomaterials (such as MXene/CNTs [89]) have been reported to be used for preparing high-performance 2D membranes. Given the effectiveness of these materials as interlayers, it is necessary to further explore the possibility of using other composite materials (such as organic polymer/1D nanomaterials, 2D/1D nanomaterials) and organic 1D nanomaterials as interlayers for preparing high-performance NF membranes. The selection of interlayer materials must also consider factors such as compatibility with the substrate, preparation difficulty, stability under operating conditions, and cost-effectiveness.
2. Current studies in membrane technology have largely focused on enhancing permeability, but there is also a pressing need for membranes that exhibit high selectivity towards specific solutes. Interlayers have shown promise in addressing this need by allowing for the customization of membrane properties to selectively adsorb or repel certain solutes, thereby enhancing selectivity. Notably, Zhu et al. [38] introduced positively charged quaternized cross-linked microgels (PNI6) as an interlayer to improve Mg^2+^ removal, demonstrating the ability of interlayers to significantly impact the selectivity of PA membranes. An exciting avenue for future research is exploring how the unique chemical properties of interlayer materials can be harnessed to enhance selectivity towards specific pollutants [90,91,92].
3. Another important aspect of developing interlayer-based TFC NF membranes is the need for scalable and cost-effective manufacturing methods. While many promising results have been reported in the literature, most of these methods are still confined to the laboratory scale and may not be suitable for large-scale production. Therefore, further research is necessary to develop scalable methods that can be seamlessly integrated into existing membrane manufacturing processes.
4. Moreover, it is crucial to conduct comprehensive investigations into the long-term stability and durability of interlayer-based TFC NF membranes under diverse operating conditions. Although several studies have reported positive outcomes in terms of membrane performance, few have explored the impact of contamination, chemical degradation, and other factors on membrane performance over extended periods of use. Hence, more extensive research is necessary to evaluate the effects of these factors on membrane performance over time.
5. Current studies on the preparation of nanofiltration membranes with an interlayer primarily focus on achieving a balance between permeability and selectivity, and there is limited research on the interlayer’s impact on anti-pollution properties. However, the impact of the interlayer on anti-fouling performance is essential in the development of nanofiltration membranes. Moving forward, we expect that further research in this area will lead to the development of novel interlayer materials and strategies that can improve both permeability/selectivity and pollution resistance simultaneously.
6. During the preparation of TFC NF membranes, controlling the dispersion of nanomaterials is crucial to avoid aggregation and ensure optimal performance. Existing literature has used various methods such as surface modification (carboxylation treatment of MWCNTs [16]), dispersion in solutions such as PDA [54,56], polyvinyl alcohol sodium [69], dodecyl benzene sulfonate [51], sodium dodecylbenzene sulfonate [56], Tris-HCl buffer [47], and ultrasound treatment [21,47,51,52,53,54,56,69,72,75,87] to prevent the agglomeration of nanomaterials. While there are many methods available to avoid the agglomeration of nanomaterials, there is always room for improvement and the development of new methods. This is because the properties and behavior of nanomaterials can be complex and difficult to predict, and different applications may require different strategies for preventing agglomeration. Additionally, as new types of nanomaterials are developed and used in various applications, new methods for avoiding agglomeration may need to be developed as well. Therefore, continued research and development in this area is necessary to optimize the performance of nanomaterials in various applications.
7. In conclusion, while there are significant challenges in the application of interlayer materials in the preparation of TFC PA NF membranes, this field holds enormous potential for enhancing membrane performance and extending its scope of applications. Through sustained research and development in this area, we may witness substantial progress in membrane technology in the forthcoming years.

## Figures and Tables

**Table 1:** Structure and performance of TFC NF membranes with organic interlayer.

Category	Organic Material	Porous Substrate	IP Condition (Optimum)	Polyamide Thickness (nm)	Water Flux (L m^−2^ h^−1^ bar^−1^)	Salt Rejection	Year [Ref]
Polyphenols	TA-Fe nano scaffold	PSF UF substrate (Mw 35,000)	0.2 wt% PIP/water, 0.15 wt% TMC/n-hexane 1 min reaction	54.9	19.6	20.6 ± 2.8 α (NaCl/MgSO_4_)	2018 [32]
	Noria–PEI solution	PSF UF substrate	1 wt% PIP/water, 0.1 wt% TMC/n-hexane 30 s reaction	39.7	28	>96% Na_2_SO_4_	2018 [25]
	TTSBI-PEI solution	PSF UF substrate(Mw 70,000)	1 wt% PIP/water, 0.1 wt% TMC/n-hexane 30 s reaction	28.6	23.7	99.4% Na_2_SO_4_	2020 [34]
	PDA	PSF substrate (Mw 35,000)	0.2 wt% PIP/water, 0.15 wt% TMC/n-hexane 1 min reaction	116	14.8	34.4 ± 1.0 α (NaCl/MgSO_4_)	2020 [20]
	TA-DDS	PSF UF substrate	0.1 *w/v*% PIP/water, 0.1 wt% TMC/n-hexane 25 s reaction	42	17.4	>99% Na_2_SO_4_	2021 [31]
	Catechol-SA	PSF UF substrate	1 mg/mL PIP/water 0.1 mg/mL TMC/n hexane 30 s reaction	110	13.71	99.15% Na_2_SO_4_	2021 [35]
	Catechol-PEI	PSF UF substrate	10 mg/mL PIP/water 1 mg/mL TMC/n hexane 30 s reaction	35	18.4	50.7 α(Mg^2+^/Li^+^)	2022 [33]
Ionic polymers	PSS-Ca^2+^	PSF UF substrate	1 *w*/*v*% PIP/water, 0.1 wt% TMC/n-hexane 30 s reaction	38.4	22.15	99.3% Na_2_SO_4_	2020 [36]
	P[MPC-co-AEMA]-GA	PSF substrate	0.2 *w*/*v*% PIP/water, 0.1 wt% TMC/n-hexane 30 s reaction	12	20.4	96.8% Na_2_SO_4_	2022 [40]
	SPEK-C	PTFE MF substrate (0.22 μm)	0.1 wt% PIP/water, 0.025 *w*/*v*% TMC/n-hexane 1 min reaction	25	36.3	98.5% Na_2_SO_4_	2022 [37]
	Quaternized crosslinked microgels	PAN UF substrate (Mw 50,000)	1.5 wt% PIP/water, 0.1 wt% TMC/n-hexane 30 s reaction	44	10.1	93.4% Na_2_SO_4_	2023 [38]
	PAH-GA	PSF UF substrate (Mw 600,000)	0.1 wt% PIP/water, 0.05 wt% TMC/n-hexane30 s reaction	8	12.5	98.9% Na_2_SO_4_	2023 [39]
Polymeric organic acid	Hyaluronic acid	PES MF substrate (0.10 μm)	0.5 wt% PIP/water, 0.1 wt% TMC/n-hexane 30 s reaction	41.5	29.53	94.9% Na_2_SO_4_	2022 [41]
	Poly(caffeic acid)	PAN UF substrate (Mw 80,000)	NA	284	17.7	98.5% Na_2_SO_4_	2023 [42]
Other organic interlayers	PVA-GA	PES UF substrate(Mw 150,000)	0.5 wt% PIP/water, 0.1 wt% TMC/n-hexane 30 s reaction	9.6	31.4	99.4% Na_2_SO_4_	2020 [27]
	Gelatin-GA	PSF UF substrate	1 *w/v*% PIP/water, 0.1 wt% TMC/n-hexane 30 s reaction	45	16.95	99.3% Na_2_SO_4_	2021 [43]
	Poly (amidoxime)	PES UF substrate (Mw 58,000)	0.2 wt% PIP/water, 0.1 wt% TMC/n-hexane 1 min reaction	18.2	25.2	99.2% Na_2_SO_4_	2022 [44]
	PEI/DNPs-GA	PSF UF substrate	1 wt% PIP/water, 0.1 wt% TMC/n-hexane 30 s reaction	33.3	31.33	99.1% Na_2_SO_4_	2022 [45]

## Data Availability

Not applicable.

## References

[1] Dai R., Li J., Wang Z. (2020). Constructing interlayer to tailor structure and performance of thin-film composite polyamide membranes: A review. Colloid Interface Sci..

[2] Park H.B., Kamcev J., Robeson L.M., Elimelech M., Freeman B.D. (2017). Maximizing the right stuff: The trade-off between membrane permeability and selectivity. Science.

[3] Yang Z., Guo H., Tang C.Y. (2019). The upper bound of thin-film composite (TFC) polyamide membranes for desalination. J. Membr. Sci..

[4] Lau W.J., Gray S., Matsuura T., Emadzadeh D., Chen J.P., Ismail A.F. (2015). A review on polyamide thin film nanocomposite (TFN) membranes: History, applications, challenges and approaches. Water Res..

[5] Lau W.J., Ismail A.F., Misdan N., Kassim M.A. (2012). A recent progress in thin film composite membrane: A review. Desalination.

[6] Choi W., Gu J.-E., Park S.-H., Kim S., Bang J., Baek K.-Y., Park B., Lee J.S., Chan E.P., Lee J.-H. (2015). Tailor-Made Polyamide Membranes for Water Desalination. ACS Nano.

[7] Freger V. (2003). Nanoscale heterogeneity of polyamide membranes formed by interfacial polymerization. Langmuir.

[8] Freger V., Srebnik S. (2003). Mathematical model of charge and density distributions in interfacial polymerization of thin films. J. Appl. Polym. Sci..

[9] Mohammad A.W., Teow Y.H., Ang W.L., Chung Y.T., Oatley-Radcliffe D.L., Hilal N. (2015). Nanofiltration membranes review: Recent advances and future prospects. Desalination.

[10] Nowbahar A., Mansard V., Mecca J.M., Paul M., Arrowood T., Squires T.M. (2018). Measuring interfacial polymerization kinetics using microfluidic interferometry. J. Am. Chem. Soc..

[11] Liang Y., Li C., Li S., Su B., Hu M.Z., Gao X., Gao C. (2020). Graphene quantum dots (GQDs)-polyethyleneimine as interlayer for the fabrication of high performance organic solvent nanofiltration (OSN) membranes. Chem. Eng. J..

[12] Geise G.M., Paul D.R., Freeman B.D. (2014). Fundamental water and salt transport properties of polymeric materials. Prog. Polym. Sci..

[13] Liu G., Jin W., Xu N. (2015). Graphene-based membranes. Chem. Soc. Rev..

[14] Karan S., Jiang Z., Livingston A.G. (2015). Sub-10 nm polyamide nanofilms with ultrafast solvent transport for molecular separation. Science.

[15] Jimenez-Solomon M.F., Song Q., Jelfs K.E., Munoz-Ibanez M., Livingston A.G. (2016). Polymer nanofilms with enhanced microporosity by interfacial polymerization. Nat. Mater..

[16] Wu M.-B., Lv Y., Yang H.-C., Liu L.-F., Zhang X., Xu Z.-K. (2016). Thin film composite membranes combining carbon nanotube intermediate layer and microfiltration support for high nanofiltration performances. J. Membr. Sci..

[17] Zhu J., Hou J., Zhang R., Yuan S., Li J., Tian M., Wang P., Zhang Y., Volodin A., Van der Bruggen B. (2018). Rapid water transport through controllable, ultrathin polyamide nanofilms for high-performance nanofiltration. J. Mater. Chem. A.

[18] Lau W.-J., Lai G.-S., Li J., Gray S., Hu Y., Misdan N., Goh P.-S., Matsuura T., Azelee I.W., Ismail A.F. (2019). Development of microporous substrates of polyamide thin film composite membranes for pressure-driven and osmotically-driven membrane processes: A review. J. Ind. Eng. Chem..

[19] Yang X., Du Y., Zhang X., He A., Xu Z.K. (2017). Nanofiltration membrane with a mussel-inspired interlayer for improved permeation performance. Langmuir.

[20] Yang Z., Wang F., Guo H., Peng L.E., Ma X.H., Song X.X., Wang Z., Tang C.Y. (2020). Mechanistic insights into the role of polydopamine interlayer toward improved separation performance of polyamide nanofiltration membranes. Environ. Sci. Technol..

[21] Long L., Wu C., Yang Z., Tang C.Y. (2022). Carbon nanotube interlayer enhances water permeance and antifouling performance of nanofiltration membranes: Mechanisms and experimental evidence. Environ. Sci. Technol..

[22] Yang Z., Sun P.F., Li X., Gan B., Wang L., Song X., Park H.D., Tang C.Y. (2020). A Critical Review on Thin-Film Nanocomposite Membranes with Interlayered Structure: Mechanisms, Recent Developments, and Environmental Applications. Environ. Sci. Technol..

[23] Kausar A. (2018). Polymer coating technology for high performance applications: Fundamentals and advances. J. Macromol. Sci. A.

[24] Ulbricht M. (2020). Design and synthesis of organic polymers for molecular separation membranes. Curr. Opin. Chem. Eng..

[25] Zhai Z., Jiang C., Zhao N., Dong W., Lan H., Wang M., Niu Q.J. (2018). Fabrication of advanced nanofiltration membranes with nanostrand hybrid morphology mediated by ultrafast Noria–polyethyleneimine codeposition. J. Mater. Chem. A.

[26] Qiu W.-Z., Yang H.-C., Wan L.-S., Xu Z.-K. (2015). Co-deposition of catechol/polyethyleneimine on porous membranes for efficient decolorization of dye water. J. Mater. Chem. A.

[27] Zhu X., Cheng X., Luo X., Liu Y., Xu D., Tang X., Gan Z., Yang L., Li G., Liang H. (2020). Ultrathin thin-Film composite polyamide membranes constructed on hydrophilic poly(vinyl alcohol) decorated support toward enhanced nanofiltration performance. Environ. Sci. Technol..

[28] Singla R.K., Dubey A.K., Garg A., Sharma R.K., Fiorino M., Ameen S.M., Haddad M.A., Al-Hiary M. (2019). Natural Polyphenols: Chemical Classification, Definition of Classes, Subcategories, and Structures. J. AOAC Int..

[29] Yang H.C., Waldman R.Z., Wu M.B., Hou J., Chen L., Darling S.B., Xu Z.K. (2018). Dopamine: Just the Right Medicine for Membranes. Adv. Funct. Mater..

[30] Zhang X., Lv Y., Yang H.-C., Du Y., Xu Z.-K. (2016). Polyphenol coating as an interlayer for thin-film composite membranes with enhanced nanofiltration performance. ACS Appl. Mater Inter..

[31] Zhao S., Li L., Wang M., Tao L., Hou Y., Niu Q.J. (2021). Rapid in-situ covalent crosslinking to construct a novel azo-based interlayer for high-performance nanofiltration membrane. Sep. Purif. Technol..

[32] Yang Z., Zhou Z.-w., Guo H., Yao Z., Ma X.-h., Song X., Feng S.-P., Tang C.Y. (2018). Tannic acid/Fe^3+^ nanoscaffold for interfacial polymerization: Toward enhanced nanofiltration performance. Environ. Sci. Technol..

[33] Chen K., Zhao S., Lan H., Xie T., Wang H., Chen Y., Li P., Sun H., Niu Q.J., Yang C. (2022). Dual-electric layer nanofiltration membranes based on polyphenol/PEI interlayer for highly efficient Mg^2+^/Li^+^ separation. J. Membr. Sci..

[34] Sun H., Liu J., Luo X., Chen Y., Jiang C., Zhai Z., Niu Q.J. (2020). Fabrication of thin-film composite polyamide nanofiltration membrane based on polyphenol intermediate layer with enhanced desalination performance. Desalination.

[35] Tian B., Hu P., Zhao S., Wang M., Hou Y., Niu Q.J., Li P. (2021). Nanofiltration membrane combining environmental-friendly polycarboxylic interlayer prepared from catechol for enhanced desalination performance. Desalination.

[36] Hu P., Tian B., Xu Z., Niu Q.J. (2020). Fabrication of high performance nanofiltration membrane on a coordination-driven assembled interlayer for water purification. Sep. Purif. Technol..

[37] Deng M., Pei T., Ge P., Zhu A., Zhang Q., Liu Q. (2022). Ultrathin sulfonated mesoporous interlayer facilitates to prepare highly-permeable polyamide nanofiltration membranes. J. Membr. Sci..

[38] Zhu S., Dong S., Fan W., Nie J., Zhu L., Du B. (2023). Preparation of high-performance nanofiltration membranes with quaternized cross-linked microgels as intermediate layer. Desalination.

[39] Song Q., Lin Y., Zhou S., Istirokhatun T., Wang Z., Shen Q., Mai Z., Guan K., Matsuyama H. (2023). Highly permeable nanofilms with asymmetric multilayered structure engineered via amine-decorated interlayered interfacial polymerization. J. Membr. Sci..

[40] Song Q., Lin Y., Ueda T., Shen Q., Lee K.-R., Yoshioka T., Matsuyama H. (2022). A zwitterionic copolymer-interlayered ultrathin nanofilm with ridge-shaped structure for ultrapermeable nanofiltration. J. Membr. Sci..

[41] Liu M., Chen W., Fu J., Wang A., Ding M., Zhang L., Han L., Gao L. (2022). Hyaluronic acid-modified nanofiltration membrane for ultrahigh water permeance and efficient rejection of PFASs. Process Saf. Environ. Prot..

[42] Wang X.-L., Xue Y.-X., Dong S.-Q., Wang Q., Yu J.-T., Wang H.-C., Zhang H., Wang W., Wei J.-F. (2023). Poly(caffeic acid) as interlayer to enhance nanofiltration performance of polyamide composite membrane. Desalination.

[43] Lan H., Li P., Wang H., Wang M., Jiang C., Hou Y., Li P., Jason Niu Q. (2021). Construction of a gelatin scaffold with water channels for preparing a high performance nanofiltration membrane. Sep. Purif. Technol..

[44] Liu Z., An Z., Mi Z., Wang Z., Zhu Q., Zhang D., Wang J., Liu J., Zhang J. (2022). Thin-film composite nanofiltration membranes with poly (amidoxime) as organic interlayer for effective desalination. J. Environ. Chem. Eng..

[45] Chen Y., Sun H., Tang S., Feng H., Zhang H., Chen K., Li P., Niu Q.J. (2022). Nanofiltration membranes with enhanced performance by constructing an interlayer integrated with dextran nanoparticles and polyethyleneimine coating. J. Membr. Sci..

[46] Song Q., Lin Y., Ueda T., Istirokhatun T., Shen Q., Guan K., Yoshioka T., Matsuyama H. (2021). Mechanism insights into the role of the support mineralization layer toward ultrathin polyamide nanofilms for ultrafast molecular separation. J. Mater. Chem. A.

[47] Wang Y., Wang T., Li S., Zhao Z., Zheng X., Zhang L., Zhao Z. (2022). Novel Poly(piperazinamide)/poly(m-phenylene isophthalamide) composite nanofiltration membrane with polydopamine coated silica as an interlayer for the splendid performance. Sep. Purif. Technol..

[48] Han S., Mai Z., Wang Z., Zhang X., Zhu J., Shen J., Wang J., Wang Y., Zhang Y. (2022). Covalent organic framework-mediated thin-film composite polyamide membranes toward precise ion sieving. ACS Appl. Mater Inter..

[49] Zhao B., Guo Z., Wang H., Wang L., Qian Y., Long X., Ma C., Zhang Z., Li J., Zhang H. (2021). Enhanced water permeance of a polyamide thin-film composite nanofiltration membrane with a metal-organic framework interlayer. J. Membr. Sci..

[50] Irigoyen J., Laakso T., Politakos N., Dahne L., Pihlajamaki A., Manttari M., Enrique Moya S. (2016). Design and Performance Evaluation of Hybrid Nanofiltration Membranes Based on Multiwalled Carbon Nanotubes and Polyelectrolyte Multilayers for Larger Ion Rejection and Separation. Macromol. Chem. Phys..

[51] Gao S., Zhu Y., Gong Y., Wang Z., Fang W., Jin J. (2019). Ultrathin polyamide nanofiltration membrane fabricated on brush-painted single-walled carbon nanotube network support for ion sieving. ACS Nano.

[52] Park M.J., Wang C., Seo D.H., Gonzales R.R., Matsuyama H., Shon H.K. (2021). Inkjet printed single walled carbon nanotube as an interlayer for high performance thin film composite nanofiltration membrane. J. Membr. Sci..

[53] Wang J.-J., Yang H.-C., Wu M.-B., Zhang X., Xu Z.-K. (2017). Nanofiltration membranes with cellulose nanocrystals as an interlayer for unprecedented performance. J. Mater. Chem. A.

[54] Zhu Y., Xie W., Gao S., Zhang F., Zhang W., Liu Z., Jin J. (2016). Single-walled carbon nanotube film supported nanofiltration membrane with a nearly 10 nm thick polyamide selective layer for high-flux and high-rejection desalination. Small.

[55] Wang Z., Wang Z., Lin S., Jin H., Gao S., Zhu Y., Jin J. (2018). Nanoparticle-templated nanofiltration membranes for ultrahigh performance desalination. Nat.Commun..

[56] Gong G., Wang P., Zhou Z., Hu Y. (2019). New insights into the role of an interlayer for the fabrication of highly selective and permeable thin-film composite nanofiltration membrane. ACS Appl. Mater. Inter..

[57] Zhao X., Liu R. (2012). Recent progress and perspectives on the toxicity of carbon nanotubes at organism, organ, cell, and biomacromolecule levels. Environ. Int..

[58] Thompson J., Bannigan J. (2008). Cadmium: Toxic effects on the reproductive system and the embryo. Reprod. Toxicol..

[59] Wang Z.-Y., Xie F., Ding H.-Z., Huang W., Ma X.-H., Xu Z.-L. (2022). Effects of locations of cellulose nanofibers in membrane on the performance of positively charged membranes. J. Membr. Sci..

[60] Ji C., Lin C.-W., Zhang S., Guo Y., Yang Z., Hu W., Xue S., Niu Q.J., Kaner R.B. (2022). Ultrapermeable nanofiltration membranes with tunable selectivity fabricated with polyaniline nanofibers. J. Mater. Chem. A.

[61] Guo Y., Ji C., Ye Y., Chen Y., Yang Z., Xue S., Niu Q.J. (2022). High performance nanofiltration membrane using self-doping sulfonated polyaniline. J. Membr. Sci..

[62] Guo Y., Wei S., Chen Y., Ye H., Xue S., Niu Q.J. (2023). Sulfonated polyaniline interlayer with controllable doping conditions for high-performance nanofiltration. J. Membr. Sci..

[63] Han S., Zhu J., Uliana A.A., Li D., Zhang Y., Zhang L., Wang Y., He T., Elimelech M. (2022). Microporous organic nanotube assisted design of high performance nanofiltration membranes. Nat. Commun..

[64] Xue S.-M., Ji C.-H., Xu Z.-L., Tang Y.-J., Li R.-H. (2018). Chlorine resistant TFN nanofiltration membrane incorporated with octadecylamine-grafted GO and fluorine-containing monomer. J. Membr. Sci..

[65] Medhekar N.V., Ramasubramaniam A., Ruoff R.S., Shenoy V.B. (2010). Hydrogen Bond Networks in Graphene Oxide Composite Paper: Structure and Mechanical Properties. ACS Nano.

[66] Lim M.-Y., Choi Y.-S., Kim J., Kim K., Shin H., Kim J.-J., Shin D.M., Lee J.-C. (2017). Cross-linked graphene oxide membrane having high ion selectivity and antibacterial activity prepared using tannic acid-functionalized graphene oxide and polyethyleneimine. J. Membr. Sci..

[67] Wang C., Li Z., Chen J., Yin Y., Wu H. (2018). Structurally stable graphene oxide-based nanofiltration membranes with bioadhesive polydopamine coating. Appl. Surf. Sci..

[68] Aburabie J., Peinemann K.-V. (2017). Crosslinked poly(ether block amide) composite membranes for organic solvent nanofiltration applications. J. Membr. Sci..

[69] Zhang J., Li S., Ren D., Li H., Lv X., Han L., Su B. (2021). Fabrication of ultra-smooth thin-film composite nanofiltration membrane with enhanced selectivity and permeability on interlayer of hybrid polyvinyl alcohol and graphene oxide. Sep. Purif. Technol..

[70] Wang S., Mahalingam D., Sutisna B., Nunes S.P. (2019). 2D-dual-spacing channel membranes for high performance organic solvent nanofiltration. J. Mater. Chem. A.

[71] Ding L., Li L., Liu Y., Wu Y., Lu Z., Deng J., Wei Y., Caro J., Wang H. (2020). Effective ion sieving with Ti_3_C_2_T_x_ MXene membranes for production of drinking water from seawater. Nat. Sustain..

[72] Xu D., Zhu X., Luo X., Guo Y., Liu Y., Yang L., Tang X., Li G., Liang H. (2021). MXene Nanosheet templated nanofiltration membranes toward ultrahigh water transport. Environ. Sci. Technol..

[73] Yu L., Ling R., Chen J.P., Reinhard M. (2019). Quantitative assessment of the iron-catalyzed degradation of a polyamide nanofiltration membrane by hydrogen peroxide. J. Membr. Sci..

[74] Ling R., Yu L., Thi Phuong Thuy P., Shao J., Chen J.P., Reinhard M. (2018). Catalytic effect of iron on the tolerance of thin-film composite polyamide reverse osmosis membranes to hydrogen peroxide. J. Membr. Sci..

[75] Zhu X., Zhang X., Li J., Luo X., Xu D., Wu D., Wang W., Cheng X., Li G., Liang H. (2021). Crumple-textured polyamide membranes via MXene nanosheet-regulated interfacial polymerization for enhanced nanofiltration performance. J. Membr. Sci..

[76] Cao S., Deshmukh A., Wang L., Han Q., Shu Y., Ng H.Y., Wang Z., Lienhard J.H. (2022). Enhancing the permselectivity of thin-film composite membranes interlayered with MoS2 nanosheets via precise thickness control. Environ. Sci. Technol..

[77] Aydiner C. (2015). A model-based analysis of water transport dynamics and fouling behaviors of osmotic membrane. Chem. Eng. J..

[78] Wang A., Xu H., Fu J., Lin T., Ma J., Ding M., Gao L. (2022). Enhanced high-salinity brines treatment using polyamide nanofiltration membrane with tunable interlayered MXene channel. Sci. Total Environ..

[79] Fu J., Xu H., Lin T., Wang A., Wang A., Yao C., Chen W., Ding M., Geng C., Gao L. (2023). Tailoring the crumpled structures of a polyamide membrane with a heterostructural MXene-TiO_2_ interlayer for high water permeability. Desalination.

[80] Wang F., Yang C., Duan M., Tang Y., Zhu J. (2015). TiO_2_ nanoparticle modified organ-like Ti_3_C_2_ MXene nanocomposite encapsulating hemoglobin for a mediator-free biosensor with excellent performances. Biosens. Bioelectron..

[81] Gao W., Li X., Luo S., Luo Z., Zhang X., Huang R., Luo M. (2021). In situ modification of cobalt on MXene/TiO_2_ as composite photocatalyst for efficient nitrogen fixation. J. Colloid Interf. Sci..

[82] Jin Y., Hu Y., Zhang W. (2017). Tessellated multiporous two-dimensional covalent organic frameworks. Nat. Rev. Chem..

[83] Biswal B.P., Chandra S., Kandambeth S., Lukose B., Heine T., Banerjeet R. (2013). Mechanochemical Synthesis of Chemically Stable Isoreticular Covalent Organic Frameworks. J. Am. Chem. Soc..

[84] Wu M., Yuan J., Wu H., Su Y., Yang H., You X., Zhang R., He X., Khan N.A., Kasher R. (2019). Ultrathin nanofiltration membrane with polydopamine-covalent organic framework interlayer for enhanced permeability and structural stability. J. Membr. Sci..

[85] Yuan J., Wu M., Wu H., Liu Y., You X., Zhang R., Su Y., Yang H., Shen J., Jiang Z. (2019). Covalent organic framework-modulated interfacial polymerization for ultrathin desalination membranes. J. Mater. Chem. A.

[86] Cheng P., Liu Y., Wang X., Fan K., Li P., Xia S. (2022). Regulating interfacial polymerization via constructed 2D metal-organic framework interlayers for fabricating nanofiltration membranes with enhanced performance. Desalination.

[87] Kang X., Liu X., Liu J., Wen Y., Qi J., Li X. (2020). Spin-assisted interfacial polymerization strategy for graphene oxide-polyamide composite nanofiltration membrane with high performance. Appl. Surf. Sci..

[88] Xue S., Lin C.W., Ji C., Guo Y., Liu L., Yang Z., Zhao S., Cai X., Niu Q.J., Kaner R.B. (2022). Thin-film composite membranes with a hybrid dimensional titania interlayer for ultrapermeable nanofiltration. Nano Lett..

[89] Ding M., Xu H., Chen W., Kong Q., Lin T., Tao H., Zhang K., Liu Q., Zhang K., Xie Z. (2020). Construction of a hierarchical carbon nanotube/MXene membrane with distinct fusiform channels for efficient molecular separation. J. Mater. Chem. A.

[90] Guo H., Deng Y., Tao Z., Yao Z., Wang J., Lin C., Zhang T., Zhu B., Tang C.Y. (2016). Does Hydrophilic Polydopamine Coating Enhance Membrane Rejection of Hydrophobic Endocrine-Disrupting Compounds?. Environ. Sci. Technol. Lett..

[91] Guo H., Yao Z., Yang Z., Ma X., Wang J., Tang C.Y. (2017). A One-Step Rapid Assembly of Thin Film Coating Using Green Coordination Complexes for Enhanced Removal of Trace Organic Contaminants by Membranes. Environ. Sci. Technol..

[92] Guo H., Deng Y., Yao Z., Yang Z., Wang J., Lin C., Zhang T., Zhu B., Tang C.Y. (2017). A highly selective surface coating for enhanced membrane rejection of endocrine disrupting compounds: Mechanistic insights and implications. Water Res..

